# Combined pre‐conditioning with salidroside and hypoxia improves proliferation, migration and stress tolerance of adipose‐derived stem cells

**DOI:** 10.1111/jcmm.15598

**Published:** 2020-08-07

**Authors:** Yuan He, Mudi Ma, Yiguang Yan, Can Chen, Hui Luo, Wei Lei

**Affiliations:** ^1^ Laboratory of Cardiovascular Diseases Guangdong Medical University Zhanjiang China; ^2^ Cardiovascular Medicine Center Affiliated Hospital of Guangdong Medical University Zhanjiang China; ^3^ Southern Marine Science and Engineering Guangdong Laboratory‐Zhanjiang The Marine Biomedical Research Institute Guangdong Medical University Zhanjiang China

**Keywords:** adipose‐derived stem cells, hypoxia, oxidative stress, salidroside, stem cell function

## Abstract

Oxidative stress after ischaemia impairs the function of transplanted stem cells. Increasing evidence has suggested that either salidroside (SAL) or hypoxia regulates growth of stem cells. However, the role of SAL in regulating function of hypoxia‐pre–conditioned stem cells remains elusive. Thus, this study aimed to determine the effect of SAL and hypoxia pre‐conditionings on the proliferation, migration and tolerance against oxidative stress in rat adipose‐derived stem cells (rASCs). rASCs treated with SAL under normoxia (20% O_2_) or hypoxia (5% O_2_) were analysed for the cell viability, proliferation, migration and resistance against H_2_O_2_‐induced oxidative stress. In addition, the activation of Akt, Erk1/2, LC3, NF‐κB and apoptosis‐associated pathways was assayed by Western blot. The results showed that SAL and hypoxia treatments synergistically enhanced the viability (fold) and proliferation of rASCs under non‐stressed conditions in association with increased autophagic flux and activation of Akt, Erk1/2 and LC3. H_2_O_2_‐induced oxidative stress, cytotoxicity, apoptosis, autophagic cell death and NF‐κB activation were inhibited by SAL or hypoxia, and further attenuated by the combined SAL and hypoxia pre‐treatment. The SAL and hypoxia pre‐treatment also enhanced the proliferation and migration of rASCs under oxidative stress in association with Akt and Erk1/2 activation; however, the combined pre‐treatment exhibited a more profound enhancement in the migration than proliferation. Our data suggest that SAL combined with hypoxia pre‐conditioning may enhance the therapeutic capacity of ASCs in post‐ischaemic repair.

## 1 INTRODUCTION

Adipose‐derived stem cells (ASCs) can be primarily harvested from adipose tissues by using a simple, minimally invasive method, and are easily cultured and expanded in vitro.^1^ A number of studies have revealed that ASCs have multi‐lineage potential as they can differentiate into multiple types of somatic cells, such as nerve cells, bone cells, endothelial cells and cardiomyocytes, under specific conditions.^2^, ^3^ Due to the cellular plasticity, ASCs are considered a promising cell source for regenerative medicine.

Transplantation of ASCs has been applied to the studies of post‐ischaemic repair.^4^, ^5^, ^6^ However, oxidative stress after ischaemia results in the production of reactive oxygen species (ROS) and inflammatory factors,^7^, ^8^ which can cause dysfunction of transplanted stem cells.^9^, ^10^, ^11^ The accumulation of ROS also contributes to stem cell ageing and various types of cell death including apoptosis, necrosis and autophagic cell death (ACD; also known as type 2 programmed cell death [PCD]). Thus, enhancing the tolerance of ASCs against oxidative injury is critical to cell transplantation. Besides, generation of a sufficient number of transplanted cells can also increase the efficiency of post‐implantation survival and proliferation capacity of stem cells in vivo.^12^

Autophagy is an evolutionarily conserved catabolic process that decomposes cytosolic proteins and organelles by forming autophagosomes to load cargo and subsequently fuse with lysosomes.^13^ Accumulating evidence has demonstrated that autophagy plays a cytoprotective role in response to cellular stress. Specifically, Liu et al reported that autophagy actually promotes hypoxia pre‐conditioning improving the viability of marrow mesenchymal stem cells (MSCs).^14^ However, excessive autophagy may lead to ACD.^15^ Several studies revealed that autophagy is involved in the regulatory mechanism of stem cell death and survival under stressed conditions.^16^, ^17^, ^18^, ^19^, ^20^, ^21^

Hypoxia (1%‐5% O_2_) pre‐conditioning is a promising strategy to optimize or increase the self‐renewal efficacy of MSCs, including bone marrow mesenchymal stem cells (BMSCs) and ASCs,^22^, ^23^ indicating that hypoxia pre‐conditioning could be an approach to increase cell yield for clinical‐scale ASC expansion. Moreover, hypoxia pre‐conditioning enhances the survival of BMSCs in ischaemic tissues by increasing autophagy and decreasing apoptosis, suggesting that hypoxia may provide a protective effect on stressed injury in MSCs.^14^ A similar stimulatory effect of hypoxia pre‐conditioning was observed on BMSC survival in vivo, with about 5% of the transplanted BMSCs remaining alive on day 14, which implies that there is still a great room to improve stem cell function under stressed or pathological conditions. Salidroside (SAL) one of the main effective constituents of traditional Chinese herb *Rhodiola* possesses diverse pharmacological effects.^25^ Indeed, SAL can promote the proliferation, differentiation, anti‐apoptosis, anti‐oxidation and anti‐inflammation activities of MSCs.^26^, ^27^, ^28^, ^29^ Therefore, SAL may further enhance the function of hypoxia‐pre–conditioned MSCs.

In this study, we determined the roles of SAL pre‐conditioning on hypoxia‐mediated proliferation and migration of rat ASCs (rASCs) by detecting the cell viability, cell proliferation, migratory ability and the activation of Akt, Erk1/2 and LC3. Furthermore, we also determined whether H_2_O_2_‐mediated cytotoxicity, cell death, redox disequilibrium and NF‐κB activation contribute to the resistance of pre‐conditioned rASCs against oxidative stress.

## 2 MATERIALS AND METHODS

### 2.1 **Culture**,** identification and transfection of rASCs**

rASCs were purchased from Cyagen Biosciences Inc. rASCs were planted in a 75‐cm^2^ culture flask and maintained in basal medium, supplemented with 10% foetal bovine serum, 2 mmol/L glutamine and 1% penicillin‐streptomycin solution. The cells were incubated in a humidified incubator with 5% CO_2_ at 37°C. The culture media were changed every two days, and the adherent cells were passaged at a confluency of approximately 80%.

P5‐7 rASCs used in this study were identified by immunophenotyping and directed differentiation of specific lineages. rASCs for immunophenotyping by flow cytometry (FCM) were digested and resuspended in 100 μL antibody working solution (90 µL of PBS containing 5% FBS and 10 µL of fluorescein‐conjugated monoclonal antibody or isotype control). The antibodies and isotype controls used for immunophenotyping were as follows: PE hamster anti‐rat CD29, PE hamster IgM, PE mouse anti‐rat CD45 and PE mouse IgG1, from BD Bioscience; CD90 monoclonal antibody (OX‐7), PE and CD34 monoclonal antibody (QBEND/10), PE, from Thermo Fisher Scientific. After being incubated in the dark on ice with shaking for 1 hour, rASCs were washed 3 times with PBS and then analysed by FCM. rASCs for osteogenic and adipogenic differentiation were cultured in 6‐well plates and orientiatedly induced using osteogenic differentiation medium and adipogenic differentiation medium, respectively. After the induction of differentiation, rASCs were stained with Alizarin red or Oil Red O and observed under an inverted phase‐contrast microscope (Leica, DMI3000 B).

The lentivirus (LV) for *stubRFP‐sensGFP‐LC3* overexpression was purchased from GeneChem. Cell transfection was performed following the protocol provided by the manufacturer. Polybrene (5 μg/mL) was added to the medium for improving transfection efficiency.

### 2.2 SAL and hypoxia pre‐conditionings

rASCs in the logarithmic growth phase were seeded in cell culture plates at a density of 3 × 10^3^/well, followed by serum deprivation for 24 hours when cells reached confluence of 50%‐60%. In order to explore the most optimal pre‐conditioning conditions, rASCs were incubated at 37°C with different concentration of SAL (0, 25, 50, 100, 200 and 400 μmol/L, respectively) in a 5% CO_2_ incubator (Thermo Fisher Scientific, 371, USA) or in a tri‐gas incubator (Thermo Fisher Scientific, 3131, USA) that maintains a 5% O_2_ level. rASCs were pre‐conditioned for 1, 3 and 5 days.

### 2.3 Cell viability analysis

The cell viability was measured using an enhanced Cell Counting Kit‐8 (CCK‐8, Beyotime). Briefly, 100 μL CCK‐8 solution was added to each well. After 2 hours of incubation at 37°C, the optical density (OD) value was measured at A_450_ nm. Three independent experiments were run.

### 2.4 Cell proliferation detection

Cell proliferation was assessed by detection of BrdU incorporation. BrdU can be inserted into the DNA chain which is replicating and thus may be used as a measure of cell proliferation. Cells grown on 13‐mm round coverslips were incubated with a final concentration of 10 μmol/L of BrdU (5‐bromo‐2'‐deoxyuridine). After 1 hour of culture, cells were fixed with 4% paraformaldehyde for 30 minutes at room temperature. The coverslips were washed with PBS and then incubated for 30 minutes with 0.1% Triton X‐100 to permeablize the membranes. Cultures were incubated with 1 mol/L HCl for 10 minutes on ice, 2 mol/L HCl for 10 minutes at room temperature and with 2 mol/L HCl for 20 minutes at 37°C. After DNA denaturation, cells were incubated with 0.1 mol/L sodium‐borate buffer, pH 8.4, for 12 minutes at room temperature, then washed three times with 0.1% Triton X‐100, blocked with 2% BSA in PBS for 1 hour at room temperature and incubated with anti‐BrdU antibodies (Cell Signaling Technology, CST) overnight at 4°C. After being washed with PBS, cells were incubated with secondary antibody (anti‐mouse IgG, Alexa Fluor 488 Conjugate, CST) at a 1:500 dilution for 1 hour at 37°C. Finally, the cell nuclei were stained with DAPI (4',6‐diamidino‐2‐phenylindole). Each coverslip was observed using a laser scanning confocal microscope (Leica, TCS SP5 II) and then analysed by counting, in a blind fashion. The BrdU‐positive cells and total cells in five viewing fields were counted under a 20 × objective microscope.

### 2.5 Observation of autophagosomes and autolysosomes

rASCs transfected with *stubRFP‐sensGFP‐LC3* LV were seeded in 30‐mm glass‐bottom culture dishes. At the end of the experiments, rASCs were observed under a laser scanning confocal microscope (LSCM, Leica). The fluorescence of yellow dots (overlays of red and green channels) and red dots was observed in five viewing fields, and was counted manually by a person unfamiliar with this study. The number of autophagosomes and autolysosomes in each cell was calculated as the yellow dots and red dot, respectively.

### 2.6 Oxidative stress induced by hydrogen peroxide (H_2_O_2_) and cytotoxicity assay

Oxidative stress was induced by addition of H_2_O_2_ (400 μmol/L) in low‐glucose DMEM (Dulbecco's modified Eagle medium) supplemented with 0.1% FBS. We first performed LDH release assay to analyse H_2_O_2_‐mediated cytotoxicity. The destruction of plasma structure caused by cell death results in the release of enzymes in cytoplasm into the culture medium, including LDH, which has relatively stable enzymatic activity. LDH release was analysed using a LDH Cytotoxicity Assay Kit (Beyotime, Shanghai, China). After H_2_O_2_ treatment for 24 hours, the cell culture plates were centrifuged for 5 minutes at 400 g. Cell culture supernatant (120 μL/well) was transferred from each well to 96‐well culture plates. Cells were washed once with PBS and incubated with 150 μL LDH release reagent (1:10 dilution) at 37°C for 1 hour; then, the supernatants were also transfer to a new culture plate. 60 μL LDH test solution was added to the transferred supernatant. The plate was incubated at room temperature (22‐25°C) in the dark for 30 minutes. Finally, the absorbances were read at 490 nm using a microplate spectrophotometer (Epoch, BioTek). The percentage of LDH release was calculated using the following formula:

$$\text{LDH release}\left(\%\right)=\frac{\text{LDH in supernatant}}{\text{LDH in supernatant}\;+\;\text{intracellular LDH}}\times 100.$$

### 2.7 Cell apoptosis assay

After being incubated with H_2_O_2_ or PBS for 24 hours, cells were fixed with 4% paraformaldehyde at room temperature for 30 minutes. In Situ Cell Death Detection Kit (Roche) was used for TUNEL staining. Briefly, cells were incubated in a 0.1% Triton‐100 solution and then incubated with a TUNEL reaction mixture. Finally, nuclei were stained with 1 μg/mL DAPI. Besides, apoptosis was also detected using the Annexin‐V‐FITC Apoptosis Detection Kit (BD Pharmingen). The protocol was performed in accordance with the manufacturer's instructions. Cells were digested into single cell suspensions with EDTA‐free trypsin and then subjected to the Annexin V/PI. The stained cells were analysed by FCM in one hour after staining.

### 2.8 Malondialdehyde (MDA) and glutathione (GSH) measurement

The contents of MDA and GSH were respectively measured using a Lipid Peroxidation MDA Assay Kit and a Total Glutathione Assay Kit (Beyotime), according to the manufacturer's instruction. Cells were homogenized with PBS, and the homogenate was centrifuged at 10 000 *g* for 10 minutes. The supernatant was taken for the subsequent measurement.

For MDA measurement, 0.1 mL homogenate or standards were added to a tube, and subsequently 0.2 mL MDA test working solution was added. The mixture was heated in boiling water bath for 15 minutes, then cooled to room temperature and centrifuged at 1,000 *g* for 10 minutes. 200 μL supernatant was added to a 96‐well plate, and then, the absorbance at 532 nm was measured using a microplate spectrophotometer. For GSH measurement, 10 μL homogenate or standards, 10 μL protein removal reagent S solution and 150 μL total glutathione detection working solution were sequentially added to each well of a 96‐well plate, then mixed and incubated at room temperature for 5 minutes. 50 μL NADPH solution (0.5 mg/mL) was added to each well. After mixing, the absorbance at 412 nm was measured every 5 minutes for a total of 25 minutes. MDA or GSH content was calculated according to the standard curve.

### 2.9 Catalase (CAT) activity assay

CAT (EC 1.11.1.6) activities were determined using a Catalase Assay Kit (Beyotime), following the manufacturer's protocols, as previously described.^30^ Cells were lysed with Cell lysis buffer for Western and IP (Beyotime), and then, the cell lysates were diluted with catalase assay buffer. 40 µL supernatants were mixed with 10 µL catalase assay buffer containing 250 mmol/L H_2_O_2_. After incubation at 25°C for 3 minutes, 450 µL of reaction termination buffer was added to stop the reaction. A total of 10 µL of the mixtures were diluted 5‐fold with the catalase assay buffer. Subsequently, 10 µL of the diluted mixtures was added into a 96‐well plate and incubated with 200 µL of working colour reagent for 20 minutes. Finally, the absorbance was measured at 520 nm by a microplate spectrophotometer (Epoch, BioTek). CAT activity was calculated and expressed as U/mg protein.

### 2.10 Cell migration assay

Transwell migration assay was performed using Transwell chambers with 8‐μm filter inserts (BD Pharmingen). Cells were trypsinized and suspended in medium with 0.5% FBS. Then, cells (30 000 cells/cm^2^) were seeded in the top of the inserts and placed in the wells containing medium with 10% FBS. After incubating the cells for 12 hours at 37°C, the inserts were washed with PBS twice and cells on the upper surface of the inserts were gently removed with a cotton swab. The Transwell filters were fixed with 4% paraformaldehyde for 30 minutes, rinsed with ultra‐pure water and stained with 0.1% crystal violet for 20 minutes. The cells that had migrated to the lower surface were visualized and photographed under an inverted microscope (Leica). The migration rate was analysed using the Image‐Pro Plus 6.0 software.

### 2.11 Western blot

Cells were lysed with RIPA Buffer. Protein concentration was determined using the BCA method. Briefly, proteins (15 μg) were denatured and separated by 10% SDS‐PAGE gels and electroblotted onto PVDF membranes. The membranes were incubated with anti‐β‐actin (1:1000), anti‐Akt (1:2000), anti‐p‐Akt (Ser473, 1:2000), anti‐Erk1/2 (1:1000), anti‐p‐Erk1/2 (Thr202/Try204, 1:1000), anti‐LC3A/B (1:1000), anti‐NF‐кB‐p65 (1:1000), anti‐p‐NF‐кB‐p65 (Ser536, 1:1000), anti‐p38MAPK (1:1000), anti‐p‐p38MAPK (1:1000), anti‐capase‐9 (1:1000), anti‐cleaved‐caspase‐9 (1:500), anti‐Bcl‐2 (1:1000) and anti‐Bax (1:1000) antibodies (CST) for at least 16 hours at 4°C. The goat anti‐rabbit IgG (1:2000, CST) was incubated at room temperature for 1 hour as the secondary antibody. Immunoreactions were detected using an electrochemiluminescence (ECL) system according to the manufacturer's instructions. The grey value was calculated by the ImageJ analysis software (National Institutes of Health, USA).

### 2.12 Statistical analysis

Data were expressed as mean ± SEM for the values obtained from three independent experiments. Statistical analyses were performed by analysis of variance (ANOVA) followed by the Bonferroni multiple comparison test (GraphPad Prism 5.0). A *P*‐value < 0.05 was considered statistically significant.

## 3 RESULTS

### 3.1 SAL and hypoxia pre‐conditionings synergistically promote cell proliferation and autophagic flux in rASCs under non‐stressed conditions

**Figure 1:**
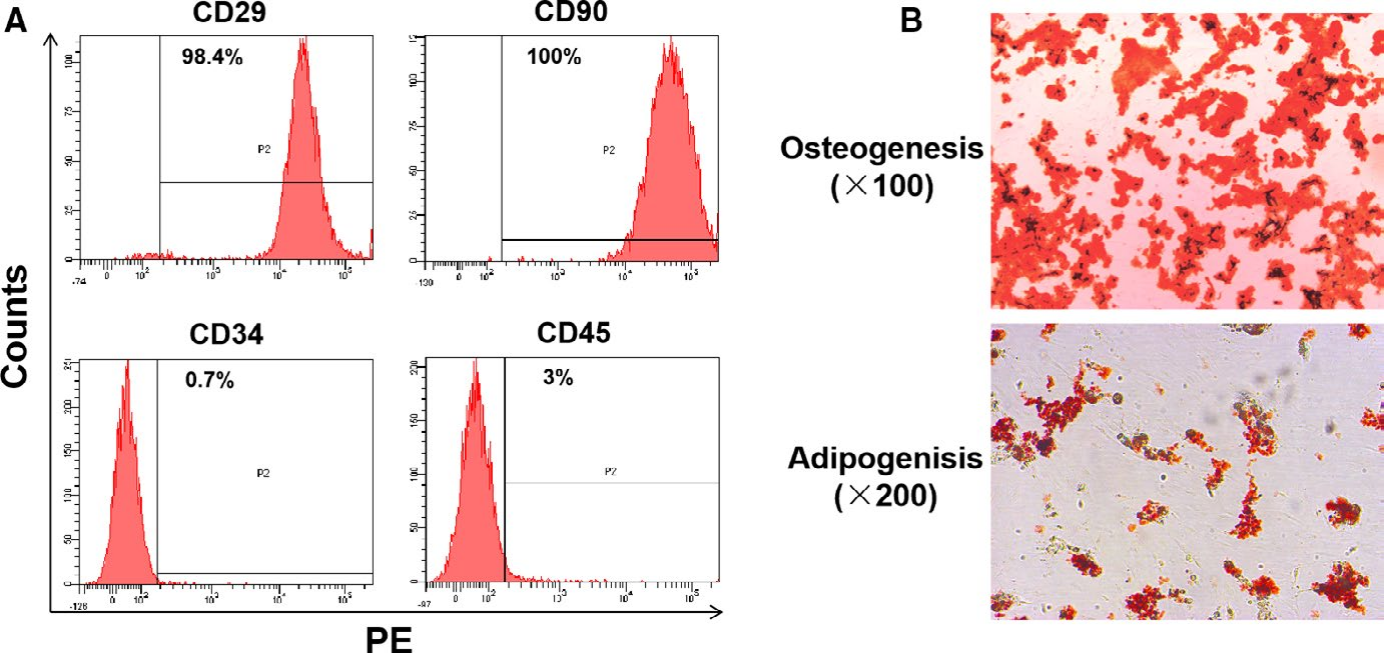
Characterization of P5‐7 rASCs. Cluster of differentiation (CD) profile of rASCs was analysed by FCM prior to treatments (A). Osteogenic‐induced and adipogenic‐induced rASCs were stained with Alizarin red and Oil Red O, respectively (B)

**Figure 2:**
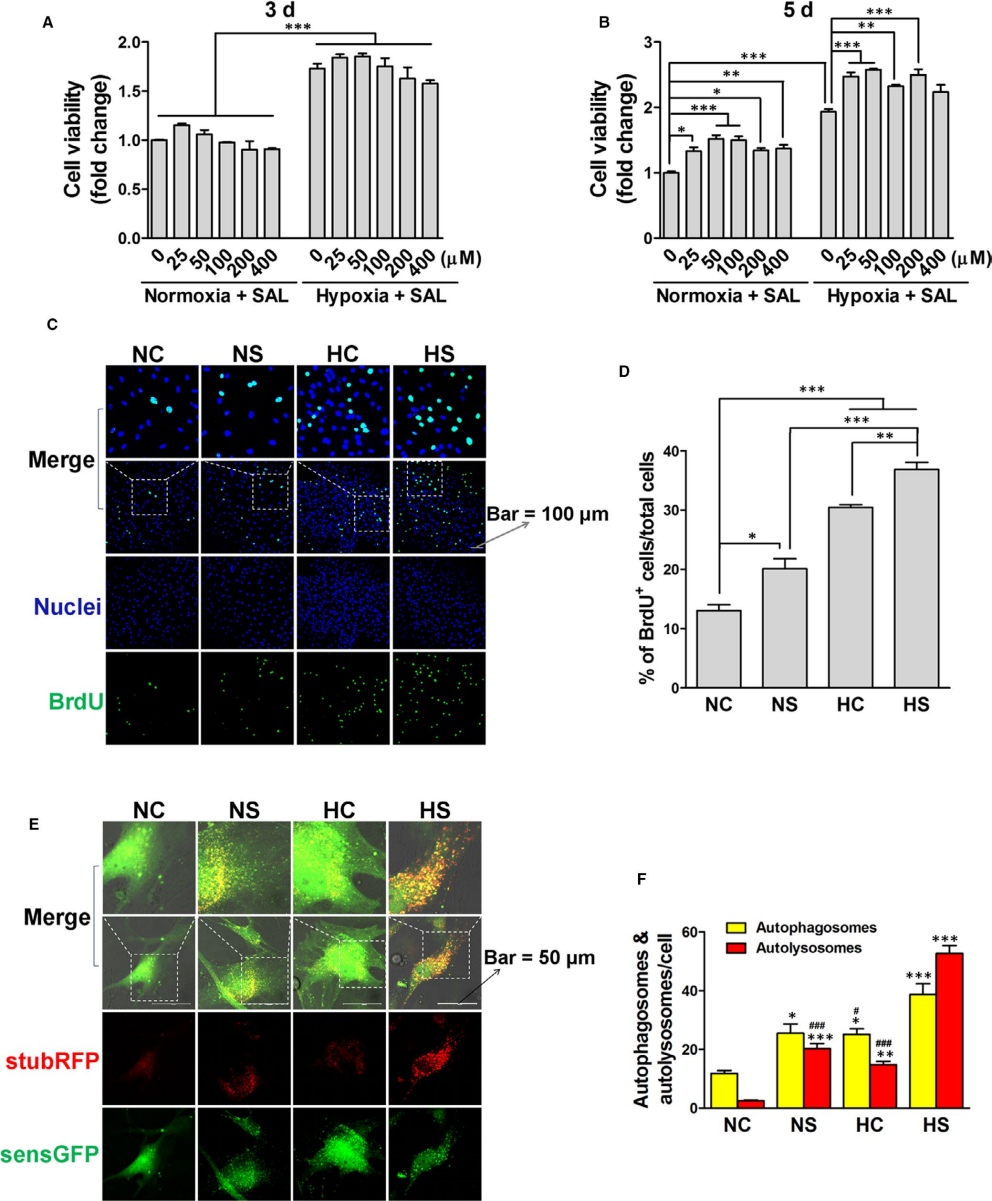
Effects of SAL and hypoxia treatments on cell viability, proliferation and autophagy. After the treatment with SAL (25‐400 μmol/L), hypoxia or hypoxia + SAL (25‐400 μmol/L), the cell viability of rASCs was determined by CCK‐8 assay on day 3 (A) and day 5 (B). Then, rASCs were treated with nomoxia + DMSO (NC), nomoxia + 50 μmol/L SAL (NS), hypoxia + DMSO (HC) or hypoxia + 50 μmol/L SAL (HS). After being treated for 5 days, the cell proliferation was analysed by BrdU incorporation assay (C and D), and the autophagic flux was assessed by stubRFP‐sensGFP‐LC3 (E and F). *P < .05, **P < .01, ***P < .001 (A, B and D). *P < .05 vs NC, **P < .01 vs NC, ***P < .001 vs NC, #*P* < .05 vs HS, ###*P* < .001 vs HS (F)

rASCs were characterized for antigens (Figure 1A) and induction of differentiation (Figure 1B). We first determined the effects of time cause of hypoxia and the dose response of SAL on cell viability of rASCs. Vehicle treatment under normoxia was used as a control. CCK‐8 assay results showed that hypoxia treatment for at least 3, but not 1 days (data not shown) significantly increased cell viability (Figure 2A,B). However, SAL did not show significant effects until day 5. Taken together, SAL at a concentration of 50 μmol/L had the most robust effects on cell viability under normoxia and hypoxia.

Based on the results of CCK‐8 assay, rASCs were treated and grouped as follows: NC group (nomoxia + DMSO), NS group (nomoxia + 50 μmol/L SAL), HC group (hypoxia + DMSO) and HS group (hypoxia + 50 μmol/L SAL). Cells in each group were treated for 5 days. The cell proliferation of rASCs was determined by the BrdU incorporation assay. To examine the status of autophagy, rASCs were transfected with *stubRFP‐sensGFP‐LC3* LV and monitored under the oil microscope. As shown in Figure 2C‐F, the NS, HC and HS groups displayed significant increases in the number of BrdU + cells and autophagosomes/autolysosomes, compared to the NC group. Moreover, the HS treatment showed synergistic effects in promoting proliferation and autophagic flux in rASCs.

**Figure 3:**
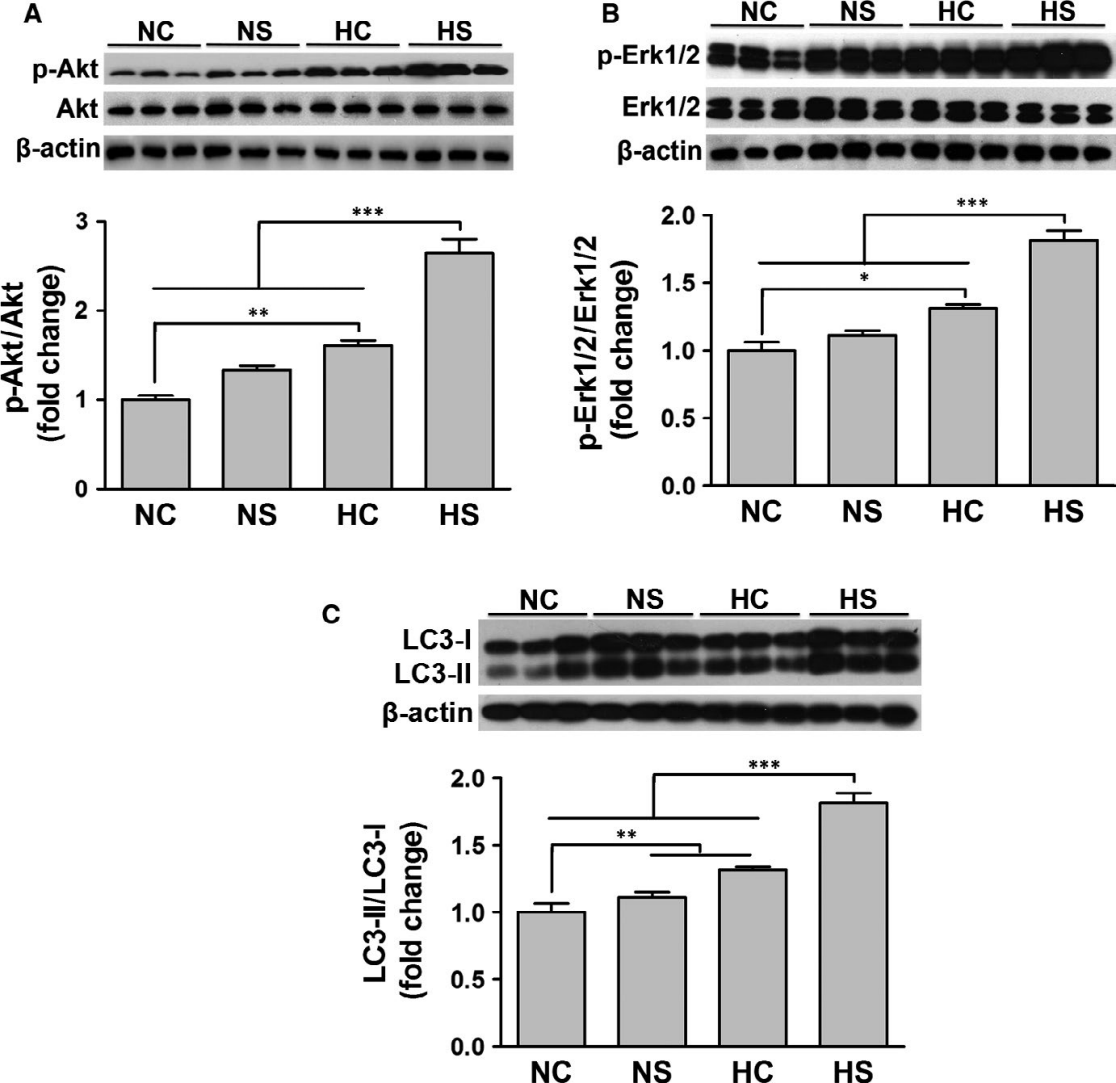
Effects of SAL and hypoxia treatments on Akt, Erk1/2 and LC3 activation. The phosphorylation levels of Akt (A) and Erk1/2 (B) and the ratio of LC3‐II/LC3‐I (C) were analysed by Western blot. *P < .05, **P < .01, ***P < .001

The expression of Akt, p‐Akt, Erk1/2, p‐Erk1/2 and LC3 was determined by Western blot. p‐Akt and p‐Erk1/2 levels were presented as the ratio of phosphorylated to total proteins. As shown in HC or HS, but not NS significant increased p‐Akt and p‐Erk1/2 (Figure 3A,B). NS, HC and HS increased the ratio of LC3‐II/LC3‐I (Figure 3C). Moreover, HS led to higher levels of p‐Akt, p‐Erk1/2 and LC3‐II/LC3‐I, compared with NS and HC. These findings indicate that SAL and hypoxia pre‐conditionings promote the proliferation of rASCs under non‐stressed conditions in association with enhanced autophagic activity and activation of Akt, Erk1/2 and LC3.

### 3.2 SAL and hypoxia pre‐conditionings synergistically attenuate H_2_O_2_‐mediated cell death

**Figure 4:**
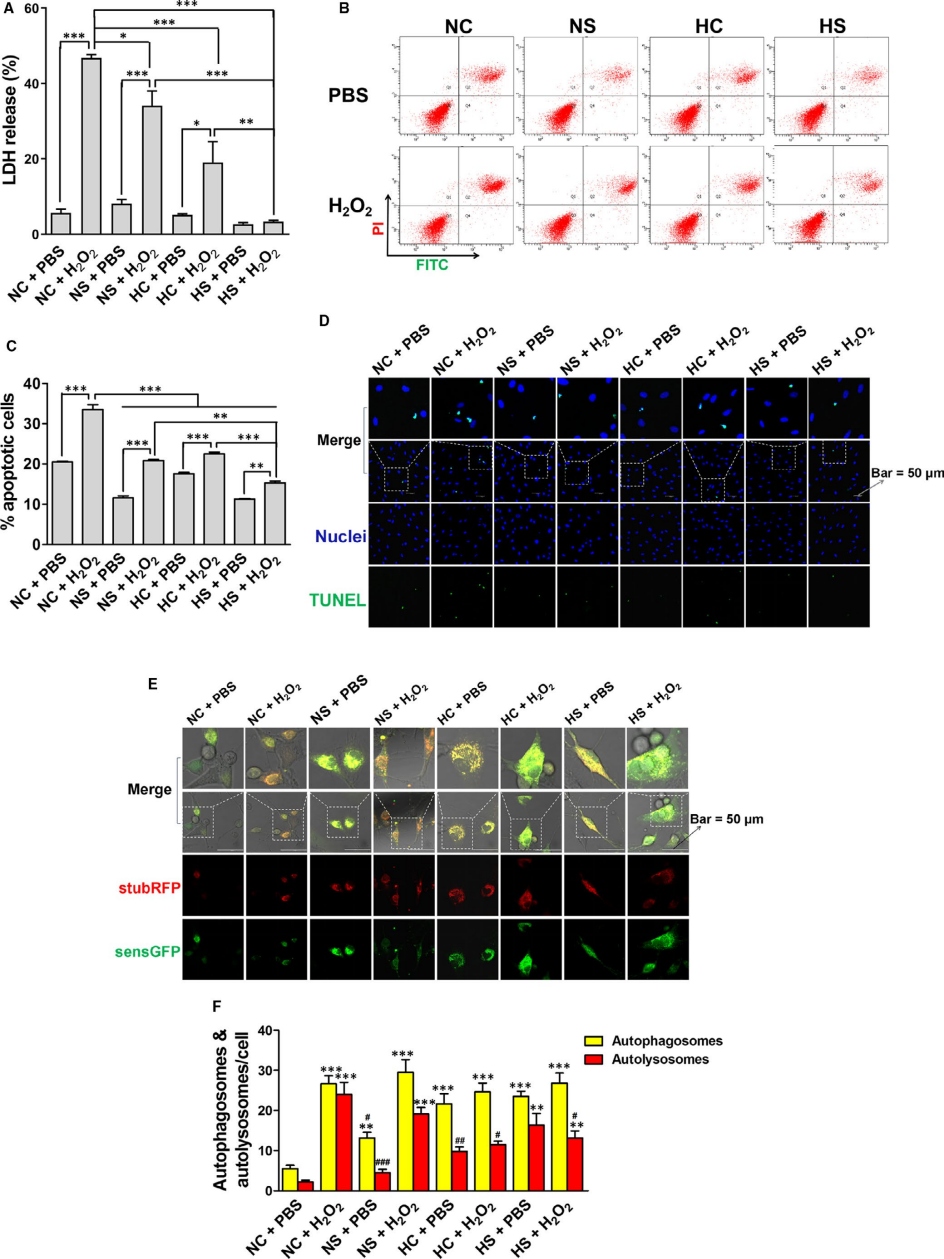
Protective effects of SAL and hypoxia pre‐treatments against H_2_O_2_‐mediated cell death. The cytotoxicity was determined by LDH release assay (A). The cell apoptosis was analysed by Annexin V‐FITC/PI‐double staining (B). The apoptosis rate was measured by calculating the percentage of late‐stage apoptotic cells (C). The apoptosis was further confirmed by TUNEL assay (D). The autophagosomes and autolysosomes were measured by stubRFP‐sensGFP‐LC3 (E), and their numbers were counted under a laser confocal microscope (F). *P < .05, **P < .01, ***P < .001 (A and C). **P < .01 vs NC + PBS, ***P < .001 vs NC + PBS, #*P* < .05 vs NC + H_2_O_2_, ##*P* < .01 vs NC + H_2_O_2_, ###*P* < .001 vs NC + H_2_O_2_ (F)

To investigate whether SAL and hypoxia pre‐conditionings have protective effects against oxidative injury, rASCs were treated with PBS or H_2_O_2_ following the NC (normoxia + DMSO), NS (normoxia + 50 μmol/L SAL), HC (hypoxia + DMSO) and HS (hypoxia + 50 μmol/L SAL) pre‐treatment. LDH release is an important indicator of cell membrane integrity and is widely used for cytotoxicity testing. As shown in Figure 4A, the NC + H_2_O_2_ group exhibited 46.8 ± 0.86% of LDH release, which was significant higher than found in the other groups. The NS, HC and HS pre‐treatments reversed LDH release caused by H_2_O_2_ treatment, and HS pre‐treatment showed a synergistic effect.

H_2_O_2_‐induced apoptosis was analysed by FCM. The NC pre‐treatment followed by the PBS treatment induced a significantly higher apoptosis, while the H_2_O_2_ treatment significantly induced a higher apoptosis rate (33.6 ± 1.62%) (Figure 4B,C). The NS and HC pre‐treatments obviously attenuated H_2_O_2_‐induced apoptosis, and the protective effects were enhanced by the HS pre‐treatment. The FCM results suggestingapoptosis were confirmed by TUNEL staining (Figure 4D). Autophagy activation in response to H_2_O_2_ stimulation was evaluated by investigating the numbers of autophagosomes and autolysosomes. We found the NC pre‐treatment followed by the H_2_O_2_ treatment resulted in significantly increased formation of autophagosomes/autolysosomes compared to the NC + PBS group, while the HC and HS pre‐treatments markedly inhibited autolysosome formation induced by H_2_O_2_ (Figure 4E,F).

**Figure 5:**
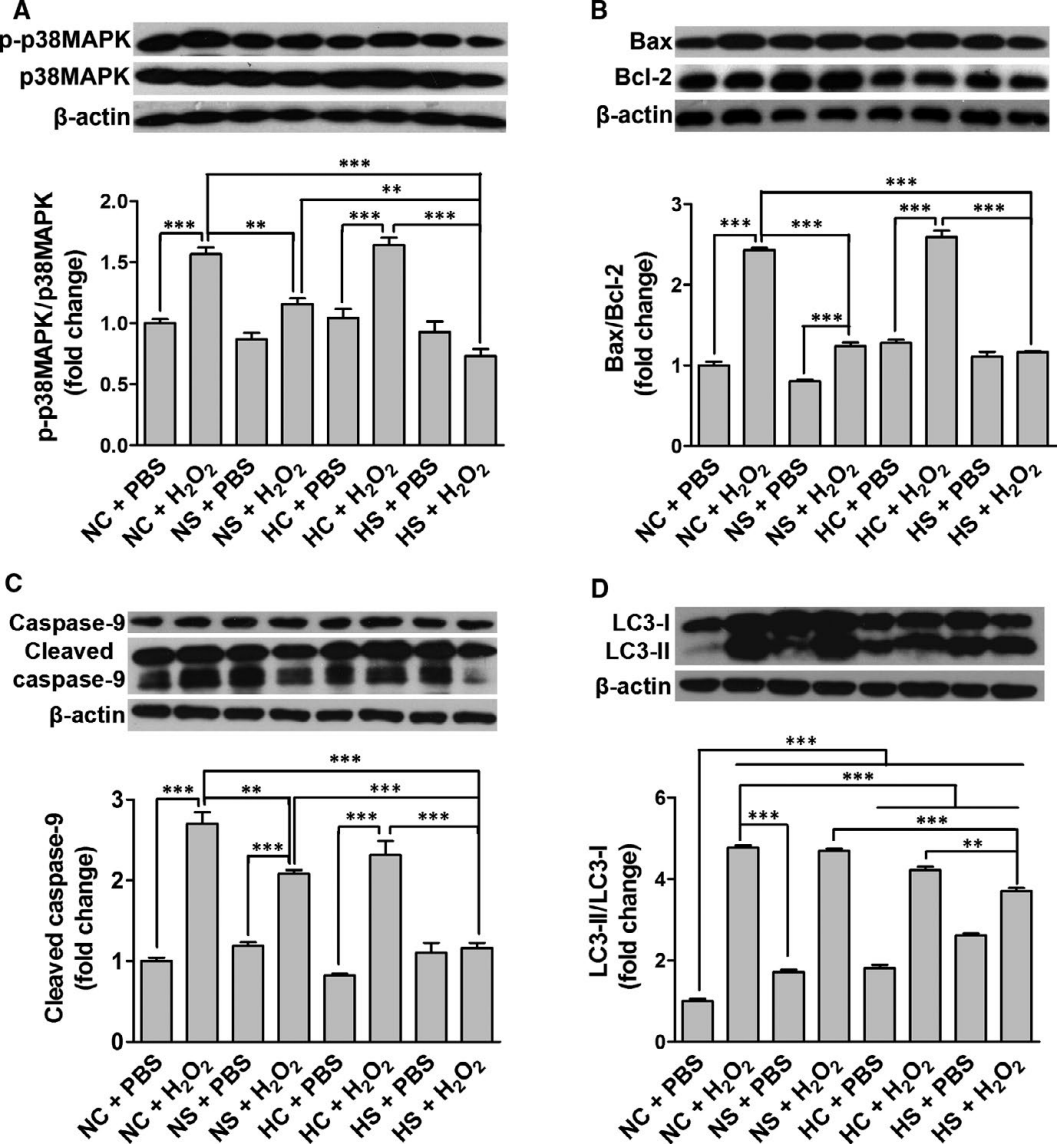
Effects SAL and hypoxia pre‐treatments on H_2_O_2_‐induced activation of apoptosis pathways and LC3. The levels of p38MAPK phosphorylation (A), Bax/Bcl‐2 (B), caspase‐9 cleavage (C) and LC3‐II/LC3‐I (D) of rASCs were determined by Western blot, after the H_2_O_2_ treatment following the NC, NS, HC or HS pre‐treatment. **P < .01, ***P < .001

To further verify the changes in cell apoptosis and autophagy, we performed Western blot to detect the ratio of Bax/Bcl‐2 and LC3‐II/LC3‐I and the levels of p‐p38MAPK and cleaved caspase‐9. We observed that p‐p38MAPK, Bax/Bcl‐2, cleaved caspase‐9 and LC3‐II/LC3‐I were significantly up‐regulated in the NC + H_2_O_2_ group, compared to the NC + PBS group (Figure 5A‐D). The NS and HS pre‐treatments significantly decreased the ratio of Bax/Bcl‐2 and the levels of p‐p38MAPK and cleaved caspase‐9, while the HC and HS pre‐treatments reduced the ratio of LC3‐II/LC3‐I (Figure 5A‐D).

Collectively, these results demonstrate that SAL and hypoxia pre‐conditionings synergistically attenuate H_2_O_2_‐mediated cell death partly by protecting rASCs against apoptosis and ACD.

### 3.3 SAL and hypoxia pre‐conditionings synergistically inhibit H_2_O_2_‐induced oxidative stress and NF‐кB activation

MDA is a natural product of lipid oxidation in organisms. Lipid oxidation occurs when oxidative stress occurs in animal or plant cells. After oxidation, fatty acids can be gradually decomposed into a series of complex compounds including MDA. Thus, the level of lipid oxidation can be accessed by detecting MDA content. GSH and CAT, the important endogenous antioxidants, can provide protection against oxidative stress by removing excessive peroxides.

**Figure 6:**
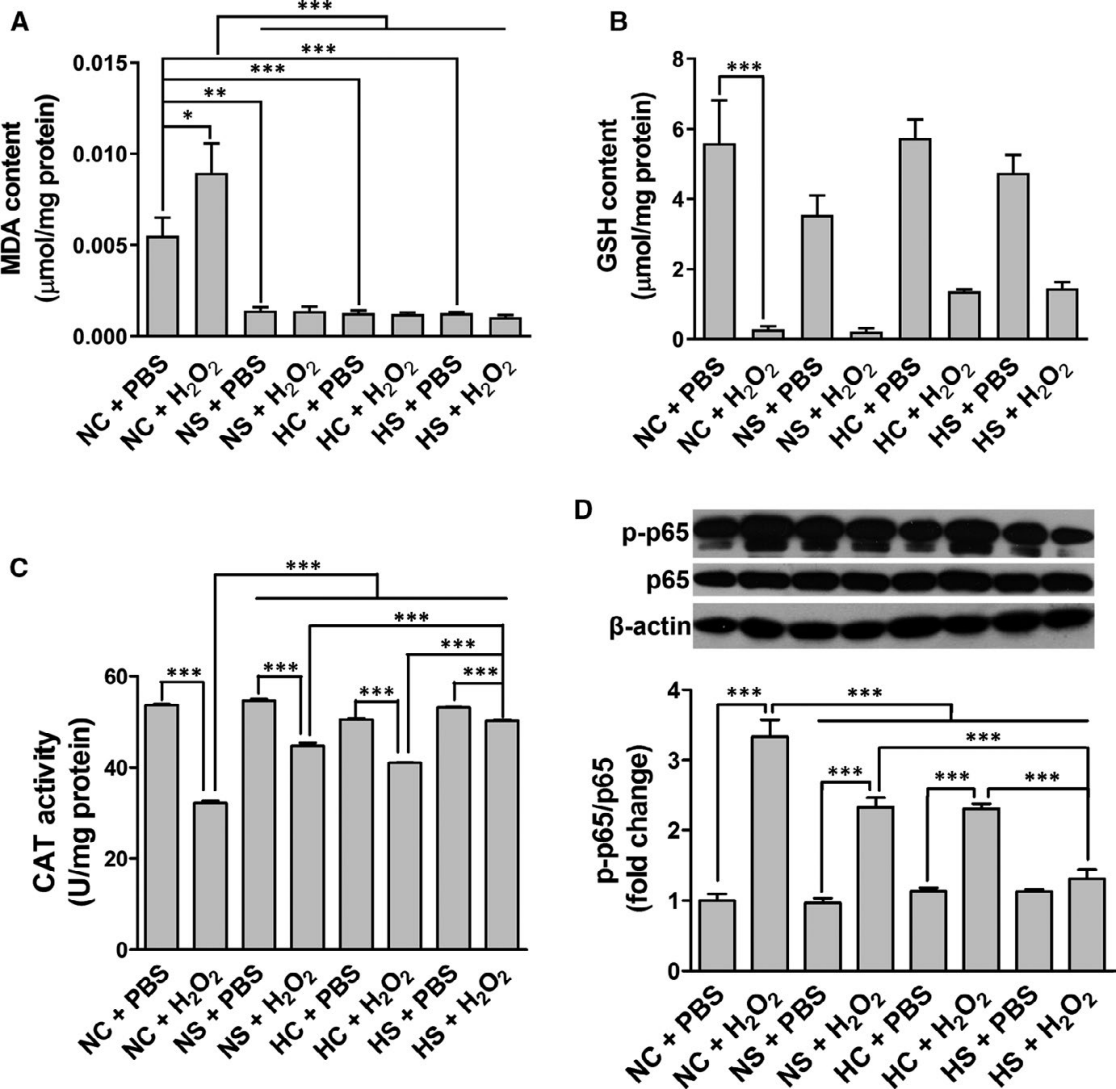
Effects of SAL and hypoxia pre‐treatments on H_2_O_2_‐induced oxidative stress and NF‐кB activation. The contents of MDA (A) and GSH (B) and the enzymatic activity of CAT (C) were quantitated in the extracts of rASCs using the colorimetric method. NF‐кB‐p65 phosphorylation (D) of rASCs was determined by Western blot. *P < .05, ***P < .001

The contents of MDA and GSH and the enzymatic activity of CAT were quantitated in the extracts of rASCs using the colorimetric method. It is found that the level of MDA was significantly up‐regulated in the NC + H_2_O_2_ group compared to other groups, suggesting that SAL and hypoxia pre‐conditionings had favourite effects on lightening lipid hyperoxidation induced by H_2_O_2_ (Figure 6A). As shown in Figure 6B,C, 24 hours of the H_2_O_2_ treatment resulted in significant reduction of GSH content and CAT enzymatic activities, while the NS, HC and HS pre‐treatments alleviated the CAT inactivation induced by H_2_O_2_, but showed no significant effects on GSH. Importantly, after the HS pre‐treatment followed by the H_2_O_2_ treatment, the changes in CAT enzyme activities showed a higher level than those in the NS + H_2_O_2_ and HC + H_2_O_2_ groups.

NF‐кB activation in response to H_2_O_2_ stimulation was evaluated by investigating the phosphorylation of NF‐кB P65. The results of Western blot showed that the H_2_O_2_ treatment significantly increased p‐NF‐кB p65 compared with the PBS treatment, after the NC, NS or HC pre‐treatment (Figure 6D). Compared with the NC + H_2_O_2_ group, rASCs pre‐treated with NS, HC or HS following by the H_2_O_2_ treatment showed significant decreases in the p‐NF‐кB p65 levels, and the HS pre‐treatment had the most obvious inhibitory efficacy (Figure 6D).

These results suggest that SAL and hypoxia pre‐conditionings synergistically protect rASCs against oxidative injury though anti‐oxidant and anti‐inflammation mechanisms.

### 3.4 SAL and hypoxia pre‐conditionings show synergistical effects on migration but not proliferation of rASCs under H_2_O_2_‐induced oxidative stress

**Figure 7:**
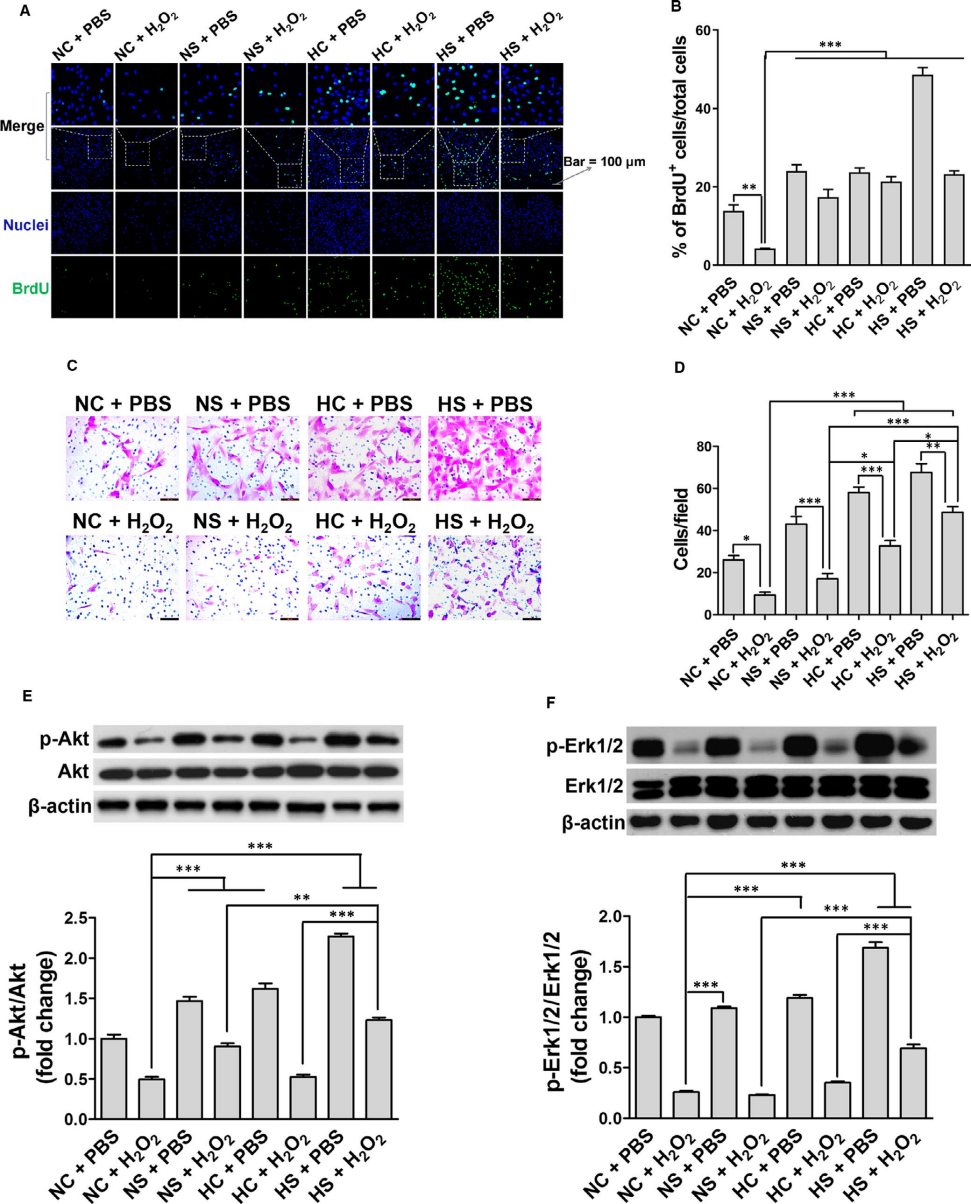
Effects of SAL and hypoxia pre‐treatments on cell proliferation, migration and activation of Akt and Erk1/2 after the H_2_O_2_ treatment. The cell proliferation was analysed by BrdU incorporation assay (A and B), and the cell migration was measured by Transwell migration assay (C and D). The phosphorylation levels of Akt (E) and Erk1/2 (F) were analysed by Western blot. *P < .05, **P < .01, ***P < .001

As shown in Figure 7A, BrdU‐positive cells represent the cells in DNA‐synthetic phase (S phase). The NS, HC and HS pre‐treatments significantly increased the proliferation of rASCs, compared to the NC + H_2_O_2_ group (Figure 7B). rASCs in the HS + PBS group showed the highest BrdU positivity ratio among each experimental group (Figure 7B).

After the NS, HC or HS pre‐treatment followed by the H_2_O_2_ treatment, the number of rASCs penetrating the Transwell chamber was respectively 1.59 ± 0.09, 1.71 ± 0.07 and 1.98 ± 0.08 folds that of the NC + H_2_O_2_ group with statistical significance (Figure 7C,D). Besides, rASCs pre‐treated with HS followed by the H_2_O_2_ treatment migrated significantly more than the NS + H_2_O_2_ and HC + H_2_O_2_ groups.

We also determined the expression of Akt, p‐Akt, Erk1/2 and p‐Erk1/2 using Western blot. The levels of p‐Akt and p‐Erk1/2 were obviously decreased after the NC pre‐treatment followed by the H_2_O_2_ treatment (Figure 7E,F). Compared to the NC + H_2_O_2_ group, NS + H_2_O_2_, HC + H_2_O_2_ and HS + H_2_O_2_ significantly increased the levels of p‐Akt and p‐Erk1/2 with HS + H_2_O_2_ group exhibiting higher levels than NS + H_2_O_2_ and HC + H_2_O_2_ groups.

In summary, these results indicate that SAL and hypoxia pre‐conditionings enhance the proliferation and migration of rASCs under oxidative stress in association with Akt and Erk1/2 activation, and SAL pre‐conditioning has a enhanced potential in improving the migration but not proliferation of hypoxia‐pre–conditioned rASCs.

## 4 DISCUSSION

This study demonstrated that SAL and hypoxia pre‐conditionings synergistically improve ASC function by modulating the proliferation, migration and tolerance against oxidative stress. rASCs cultured with SAL or 5% O_2_ exhibited significantly enhanced cell proliferation and autophagic flux, and the effects were enhanced by the combined treatment. Furthermore, H_2_O_2_‐induced cell death, excessive oxidative stress and inflammation were attenuated by the pre‐treatment with SAL, hypoxia or the both; meanwhile, late‐stage autophagy in rASCs was inhibited, and the capacity of proliferation and migration of rASCs were obviously improved. The results of Western blot revealed that Akt, Erk1/2, LC3, NF‐κB and apoptosis pathways may be involved in this regulatory mechanism.

Recent studies have revealed that low oxygen tension or hypoxia promotes the survival and function of MSCs.^14^, ^22^, ^23^, ^24^ In addition, pre‐conditioning with the components of the traditional Chinese herbs is also a promising strategy.^25^, ^26^, ^27^, ^28^, ^29^ Using the cell number counting assay and BrdU incorporation assay, we found that the treatment with SAL or hypoxia (5% O_2_) increased the proliferation capacity and autophagic flux of rASCs under non‐stressed conditions, and the combined treatment enhanced these effects (Figure 2). Moreover, up‐regulated levels of p‐Akt, p‐Erk1/2 and LC3‐II/LC3‐I were observed after the individual or combined treatment, which may explain the mechanisms of SAL and hypoxia pre‐conditionings involved in the proliferation of rASCs (Figure 3). These findings suggest that SAL pre‐conditioning combined with hypoxia pre‐conditioning allows the production of numerous rASCs from a few donor cells.

Accumulating evidence has demonstrated the poor survival and retention of transplanted cells in vivo, due to the properties of cell themselves and the extremely hostile microenvironment in the infarct tissues.^31^, ^32^ A prior study has noticed the importance that survival, proliferation and migration of implanted stem cells within the cardiac environment are crucial to the therapeutic efficacy of stem cell transplantation.^33^ Thus, extending the lifespan of stem cells is required for improving their survival under ischaemic conditions. Oxidative stress and inflammatory responses following infarction are implicated in the pathogenesis of stem cell dysfunction.^34^ Oxidative stress‐induced ROS have been shown to cause cell death and cellular ageing in different cell types.^35^, ^36^ In response to the infarct microenvironment, the stressed stem cells release various immunomodulatory signalling factors, including both pro‐inflammatory and anti‐inflammatory cytokines, thereby displaying their immunomodulatory roles.^35^, ^37^

As one of the common ROS, H_2_O_2_ can easily cross the plasma membrane and stimulate consecutive reactions leading to cell apoptosis and inflammatory responses.^36^ Our present results show that the H_2_O_2_ treatment led to increased cytotoxicity, apoptosis, late‐stage autophagy, oxidative stress and NF‐κB activation in rASCs, which were alleviated by the SAL or hypoxia pre‐treatment; moreover, the combined pre‐treatment with SAL and hypoxia has synergetic effects (Figures 4, 5, 6). The results imply that SAL pre‐conditioning combined with hypoxia pre‐conditioning can validly attenuate H_2_O_2_‐mediated cell death and inflammatory responses following the initial oxidative stress, thus may promote stem cells to exert immunosuppressive function and tissue repair activity.

In the current study, cell proliferation and migration of rASCs under H_2_O_2_‐induced oxidative stress were respectively observed by BrdU incorporation assay and Transwell migration assay. The results showed that the pre‐treatment with SAL, hypoxia or the both is key to maintaining cell proliferation and migration of rASCs under oxidative environment; the combined pre‐treatment showed the best effects on cell migration (Figure 7A‐D). Lots of evidences have shown that Akt and Erk1/2 signalling is involved in the viability, proliferation and migration of stem cells including MSCs.^38^, ^39^, ^40^, ^41^, ^42^, ^43^ Moreover, the results of Western blot confirmed that Akt and Erk1/2 signalling contributes to proliferation and migration of pre‐conditioned rASCs under oxidative stress (Figure 7E,F). These findings suggest that SAL pre‐conditioning may further facilitate the self‐renewal divisions and homing ability of hypoxia‐pre–conditioned stem cells in the ischaemic micro‐environment.

Autophagy, an intracellular degradation and recycling of cytoplasmic contents, plays a double‐edged sword effect on cell fate.^13^ Several studies have reported that autophagy is involved in the survival and proliferation of MSCs.^44^, ^45^, ^46^ However, recent reports have indicated that chronic stress and ROS can induce ACD of stem cells.^16^, ^17^, ^18^, ^19^, ^20^, ^21^ In the present study, rASCs were transfected with LV which mediated stable expression of stubRFP‐sensGFP‐LC3 in rASCs. During autophagy, LC3‐I is converted to an autophagosome‐associating form called LC3‐II.^47^ Sens‐GFP is an acid‐sensitive protein, while stub‐RFP is a stable fluorescent protein unaffected by the internal environment of lysosome. Thus, the yellow dots (overlays of red and green channels) and red dots respectively represent autophagosomes and autolysosomes. Here, we found the SAL, hypoxia and combined treatments displayed substantial increase in cell proliferation and autophagic flux in rASCs, suggesting autophagy may be beneficial to the survival and proliferation of stem cells under non‐stressed conditions (Figure 2E,F). Interestingly, we also found H_2_O_2_ induced cytotoxicity along with simultaneous up‐regulation of autophagosomes/autolysosomes in rASCs; in contrast, the hypoxia and combined pre‐treatments reversed the up‐regulation of autolysosomes under oxidative stress (Figure 4A,E,F). These findings indicate that stem cell growth and survival under non‐stressed and stressed conditions are differentially regulated by autophagy. The SAL and hypoxia treatments can facilitate cytoprotective autophagy in non‐stressed cells by up‐regulating early‐ and late‐stage autophagy, while the hypoxia and combined pre‐treatments eliminate ACD of stressed cells by attenuating the late‐stage autophagy, suggesting differential regulatory mechanisms may be involved in the process of SAL and hypoxia pre‐conditionings modulating autophagic flux under different conditions. Hence, further investigations will need to focus on the underlying mechanisms of how SAL and hypoxia pre‐conditionings counterbalance the autophagic activity of stem cells.

## 5 CONCLUSIONS

**Figure 8:**
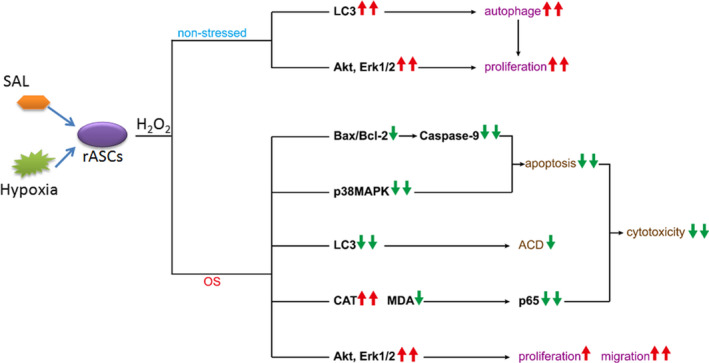
Schematic diagram demonstrates SAL pre‐conditioning combined with hypoxia improves the function of rASCs under non‐stressed and stressed conditions. OS, oxidative stress

In conclusion, our results shed light on a better understanding of the effects and mechanisms of SAL pre‐conditioning combined with hypoxia pre‐conditioning on rASC function (Figure 8). The present study clearly demonstrates for the first time that SAL pre‐conditioning further improves the function of hypoxia‐pre–conditioned rASCs by enhancing the proliferation, migration and tolerance against oxidative stress. Importantly, we partly identified the mechanisms underlying the multi‐target effects of SAL and hypoxia pre‐conditionings on rASC function. This study also suggests that SAL pre‐conditioning combined with hypoxia may facilitate a higher therapeutic capacity of ASCs in post‐ischaemic repair.

## CONFLICTS OF INTEREST

The authors declare that there is no conflict of interests regarding the publication of the paper.

## AUTHOR CONTRIBUTION

**Yuan He:** Data curation (equal); Formal analysis (equal); Methodology (equal); Writing‐original draft (equal); Writing‐review & editing (equal). **Mudi Ma:** Data curation (equal); Formal analysis (equal); Investigation (equal); Methodology (equal). **Yiguang Yan:** Data curation (equal); Methodology (equal). **Can Chen:** Funding acquisition (equal); Project administration (equal); Validation (equal). **Hui Luo:** Funding acquisition (equal); Project administration (equal). **Wei Lei:** Funding acquisition (equal); Project administration (equal); Writing‐review & editing (equal).

## Data Availability

The data that support the findings of this study are available from the corresponding author upon reasonable request.
